# The Great Gut Mimicker: A case report of MIS-C and appendicitis clinical presentation overlap in a teenage patient

**DOI:** 10.1186/s12887-021-02724-x

**Published:** 2021-06-01

**Authors:** Michelle Hwang, Kelsey Wilson, Lisa Wendt, Joshua Pohlman, Emily Densmore, Caitlin Kaeppler, Kyle Van Arendonk, Sarah Yale

**Affiliations:** Department of Pediatrics, Medical College of Wisconsin, Children’s Corporate Center Suite 560, 999 North 92nd Street, Wisconsin 53226 Milwaukee, USA

**Keywords:** Appendicitis, appendectomy, MIS-C, COVID-19, radiologic appendicitis mimic

## Abstract

**Background:**

Abdominal pain and other gastrointestinal symptoms are common presenting features of multisystem inflammatory syndrome in children (MIS-C) and can overlap with infectious or inflammatory abdominal conditions, making accurate diagnosis challenging.

**Case Presentation:**

We describe the case of a 16-year-old female who presented with clinical symptoms suggestive of appendicitis and an abdominal computed tomography (CT) that revealed features concerning for appendicitis. After laparoscopic appendectomy, histopathology of the appendix demonstrated only mild serosal inflammation and was not consistent with acute appendicitis. Her overall clinical presentation was felt to be consistent with MIS-C and she subsequently improved with immunomodulatory and steroid treatment.

**Conclusions:**

We note that MIS-C can mimic acute appendicitis. This case highlights MIS-C as a cause of abdominal imaging with features concerning for appendicitis, and MIS-C should be considered in the differential for a patient with appendicitis-like symptoms and a positive COVID-19 IgG. Lab criteria, specifically low-normal white blood cell count and thrombocytopenia, appears to be of high relevance in differing MIS-C from acute appendicitis, even when appendix radiologically is dilated.

## Background

Studies of coronavirus disease 2019 (COVID-19) in children have demonstrated an overall milder clinical course and more favorable outcomes compared to adults. [1–3] However, there is a subset of pediatric patients who develop multisystem inflammatory syndrome in children (MIS-C), a hyperinflammatory state that is temporally related to a recent infection with severe acute respiratory syndrome coronavirus 2 (SARS-CoV-2). [3] These patients may exhibit features of Kawasaki disease, signs of systemic inflammation, end organ dysfunction and shock. Laboratory features include elevated inflammatory markers, lymphopenia, neutrophilia, and thrombocytopenia. [4–6] The presence of high fever, rash, conjunctivitis, severe abdominal pain, and neck pain, with a history of SARS-CoV-2 exposure are “red flags” for MIS-C. [5].

Up to 84 % of patients with MIS-C have gastrointestinal symptoms (abdominal pain, nausea, vomiting, diarrhea) as a prominent presenting characteristic. [6, 7] Several reports describe patients with MIS-C whose presentation is concerning for a surgical diagnosis, prompting abdominal imaging and/or operative intervention. [6–9] In this case report, we describe an adolescent whose presentation with MIS-C included clinical and radiologic signs of appendicitis but had a negative appendectomy.

## Case Presentation

A previously healthy 16-year-old female presented with a four-day history of abdominal pain, vomiting, fever, headache, myalgias and cough. Her initial vital signs in the referring emergency department were temperature 39.4° Celsius, pulse 154, respiratory rate 16, blood pressure 115/61, and oxygen saturation 96 %. Physical examination was notable for pallor and right lower quadrant (RLQ) abdominal tenderness without guarding or rebound. Initial laboratory testing significant for white blood cell (WBC) count 5.8 10^3/uL (reference range 4-10.5) with 93 % neutrophils, hemoglobin 11.9 g/dL (12–15), platelets 102 10^3/uL (150–450), C-reactive protein 11 mg/dL (0–1.0), erythrocyte sedimentation rate 26 mm/hr (0–20), and procalcitonin 0.50 ng/mL (< 0.09). Urine hCG negative. Urinalysis showed trace leukocyte esterase (negative), negative nitrites, and 1–5 WBCs (0). Contrast-enhanced computed tomography (CT) of the abdomen/pelvis showed mesenteric edema, dilation of the appendix (8mm), and fat stranding throughout the lower abdomen and pelvis. She received two intravenous (IV) fluid boluses, anti-pyrectics and was transferred to our pediatric hospital.

On arrival she was evaluated by the pediatric surgery team who felt her presentation was not consistent with acute appendicitis. Further history and exam revealed that she had mild bilateral conjunctival injection and neck tenderness in addition to RLQ pain and had a positive SARS-CoV-2 polymerase chain reaction (PCR) test one month prior (she was asymptomatic but underwent testing due to several family members testing positive).

Additional labs were obtained with concern for MIS-C and were significant for a positive SARS-CoV-2 IgG antibody test and normal troponin and N-terminal Pro-Brain Natriuretic Peptide. Given fever, positive SARS-CoV-2 IgG, laboratory evidence of inflammation, and multisystem involvement she was hospitalized for further monitoring and treatment of MIS-C.

**Fig. 1:**
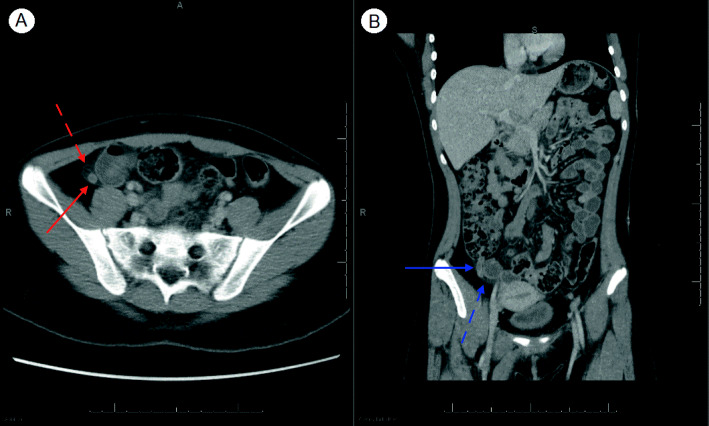
CT abdomen/pelvis. (**A**) Appendix (red solid arrow) wall thickness > 3mm. Lack of luminal air, appendix fluid depth > 2.6mm, and hyperdensity. Red dashed arrow shows peri-appendiceal fat stranding. (**B**) A diameter of 8mm (blue solid arrow) with inflammatory fat stranding (blue dashed arrow), most pronounced around the cecum

**Fig. 2:**
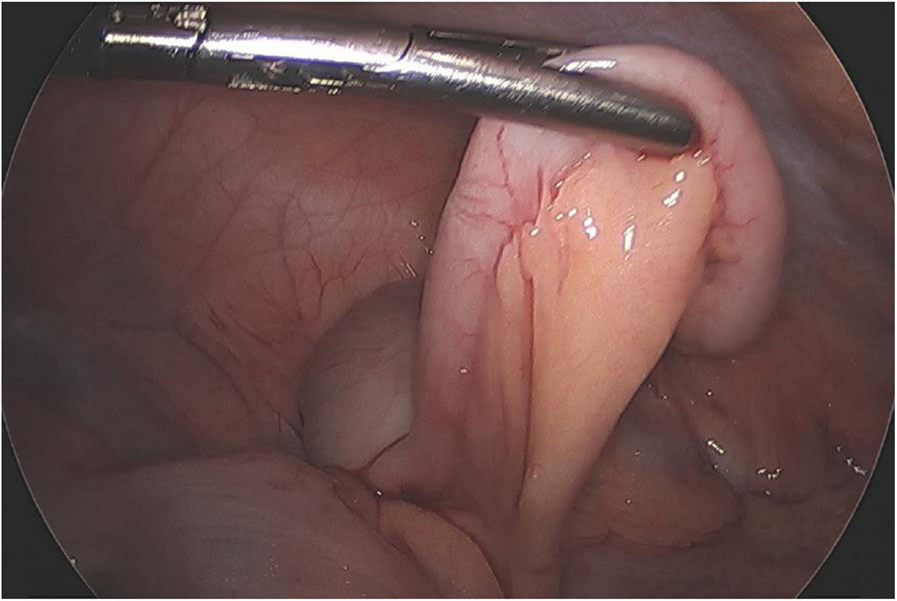
Intraoperative photograph revealing a grossly normal appendix without signs of inflammation

Overnight, the patient was persistently febrile and tachycardic. She also reported increasing RLQ pain and exhibited new abdominal rebound tenderness. The attending pediatric radiologist’s review of the prior CT concluded that the imaging was consistent with acute appendicitis as there was dilation of the appendix, measuring 8 mm, mild appendiceal mucosal hyperenhancement and adjacent mesenteric fat stranding (Fig. 1). The patient’s care was re-discussed with pediatric surgery and together the multidisciplinary team was unable to definitively rule out appendicitis as a concurrent pathology. While a diagnosis of MIS-C generally requires exclusion of other etiologies, it was felt that she could have appendicitis and MIS-C simultaneously. Empiric treatment for appendicitis was started with piperacillin-tazobactam. The surgical team reviewed the risks and benefits of non-operative management with continued antibiotics versus diagnostic laparoscopy and appendectomy. Surgical management was selected. Echocardiogram was obtained to assess for cardiac involvement and showed normal cardiac function and no dilation of the coronary arteries. She remained stable on the acute floor on hospital day two and therapy with intravenous immune globulin (IVIG) and aspirin were ordered for treatment of MIS-C. However, given the timing of when surgery could take her to the operating room, she underwent diagnostic laparoscopy and appendectomy first. She was found to have a grossly normal appendix with no inflammation to suggest appendicitis (Fig. 2). As a surgeon’s intra-operative “eyeball” assessment of appendicitis is not perfect and the risk of appendectomy while undergoing laparoscopy is low the decision was made to complete appendectomy. Histologic findings revealed only mild chronic serosal inflammation and edematous mesothelium; it did not show the transmural acute inflammation diagnostic of appendicitis. Piperacillin-tazobactam was discontinued and the patient was returned to the acute care floor for post-surgical monitoring and MIS-C treatment with IVIG infusion (2 g/kg) and aspirin (81 mg).

On hospital day three she completed IVIG therapy, and her blood pressure and fever curve improved. Repeat labs showed worsening lymphocytopenia, anemia, and thrombocytopenia and hypoalbuminemia. She then developed tachycardia and hypotension refractory to fluid resuscitation and given concern for refractory MIS-C, she was started on corticosteroid therapy (2 mg/kg twice daily). She was transferred to the intensive care unit and started on norepinephrine (0.02 mcg/kg/min) for hemodynamic support, which she required for 24 hours. The patient stabilized and was then transferred back to the acute care floor with improvement of her pancytopenia over the next two days. She was discharged home in stable condition on day seven of hospitalization to complete a course of low dose aspirin and a steroid taper.

At her follow-up cardiology appointment four weeks after discharge, she was asymptomatic, but echocardiogram showed moderately dilated right and left coronary arteries with normal ventricular function. She remained on daily aspirin with close cardiology follow up to monitor progression of her coronary involvement.

## Discussion and Conclusions

There is increasing recognition of gastrointestinal involvement in patients with COVID-19 and MIS-C. Several case reports describe patients who present with typical symptoms of appendicitis who are also found to be infected with SARS-CoV-2 virus or meet the diagnosis for MIS-C. [10–12] Studies have demonstrated that most children with MIS-C will present with gastrointestinal (GI) symptoms. [4, 7] MIS-C should be high on the differential for patients who present with GI symptoms and a history of recent SARS-CoV-2 exposure or infection, even if findings seem consistent with other GI pathologies such as appendicitis, infection, or inflammatory bowel disease (IBD).

The current case demonstrates MIS-C as a radiologic mimic of acute appendicitis on CT in a patient who underwent negative appendectomy. A prior report described a case of MIS-C that mimicked appendicitis clinically and on ultrasound imaging, prompting IV antibiotics and surgery (open appendectomy and resection of an inflamed segment of ileum) before treatment with IVIG and steroids was initiated. Pathology did not show appendicitis but did show necrotic mesenteric lymphangitis and vasculitis. [13] A South African series described four children with appendicitis, confirmed by surgical findings, in the setting of SARS-CoV-2-positive PCR. MIS-C was diagnosed in three of these children after appendectomy. [14] Other case series have described appendicitis in the setting of acute SARS-CoV-2 infection. [11, 12, 14] In addition, an increase in the rate of appendiceal perforation in children without SARS-CoV-2 infections has been found during the COVID-19 pandemic, presumably due to reluctance to present to the hospital for evaluation [15]. The hyperinflammatory state seen in COVID-19 and MIS-C may play a role in the pathogenesis of intestinal involvement. There is a similar known association with Kawasaki disease and gastrointestinal manifestations, including appendicitis. [16, 17] Others hypothesize the contributory role of the angiotensin-converting enzyme 2 (ACE2) receptor that is expressed in the intestine, especially the terminal ileum, [18] allowing SARS-CoV-2 to invade gastrointestinal cells. [11, 12].

Our patient had some findings of appendicitis with RLQ tenderness, fever, nausea, emesis, and decreased appetite. Many of the classic signs of appendicitis (fever, anorexia, nausea, guarding, and migration of pain from the umbilical region to the RLQ) may be absent in children with appendicitis; these findings also may be present with diseases other than appendicitis. [19] Up to 20 % of patients with appendicitis present without leukocytosis, [19] and our patient presented with an initially normal WBC count.

The patient’s CT had findings concerning for acute appendicitis. CT has a sensitivity of 94 % and specificity of 95 % for appendicitis. [19] Causes of false positive CT include appendiceal neoplasms, IBD, cystic fibrosis, viral infections, and tubo-ovarian infection. [20] In a study of radiologic findings in MIS-C, abdominal imaging showed small-volume ascites (38 %), hepatomegaly (38 %), echogenic kidneys (31 %), bowel wall thickening (19 %), gallbladder wall thickening (19 %), mesenteric lymphadenopathy (13 %), splenomegaly (6 %), and bladder wall thickening (16 %).[21] Appendiceal dilation and fat stranding on CT, diagnostic of appendicitis and present in our patient, have not previously been described.

Non-operative management with antibiotics alone has been quite successful in managing acute uncomplicated appendicitis meeting strict inclusion criteria[22] and was a strong consideration in this patient. However, the success of antibiotics to treat appendicitis in the setting of concurrent treatment with IVIG and steroids for MIS-C is unknown with no outcomes data currently available in the literature. Additionally, given the high likelihood that the patient’s abdominal symptoms would continue given her MIS-C (independent of the diagnosis of appendicitis), the team was concerned they would be unable to reliably follow her abdominal exam to determine if her appendicitis was being adequately treated with antibiotics.

While our patient’s clinical presentation and radiographic findings necessitated surgical evaluation, her surgical and pathologic findings ultimately were not consistent with appendicitis. Laboratory criteria may be important in helping to differentiate MIS-C from acute appendicitis - specifically, lymphopenia, thrombocytopenia, and inappropriately normal WBC count. Our patient had a progressive lymphopenia and thrombocytopenia that did not fit with the classic presentation of appendicitis.

Patients with MIS-C can present with findings characteristic of acute appendicitis, including RLQ pain, fever, nausea, emesis, anorexia, and radiographic evidence of appendicitis. This presents a diagnostic challenge for clinicians. Although difficult to exclude appendicitis in the case of CT evidence, certain laboratory criteria, namely relative leukopenia and thrombocytopenia may be helpful in differentiating these patients. However, the use of immunomodulatory agents in the treatment of MIS-C may complicate the care of a patient with potential appendicitis, and appendectomy may still be preferable as the lowest risk option despite a low clinical suspicion for appendicitis.

## Data Availability

not applicable.

## Abbreviations

MIS-C: Multi-inflammatory syndrome in children
CT: Computer tomography
COVID-19: Coronavirus disease 2019
SARS-CoV-2: Severe acute respiratory syndrome coronavirus 2
RLQ: Right lower quadrant
WBC: White blood cell
IV: Intravenous
PCR: Polymerase chain reaction
IVIG: Intravenous immune globulin
GI: Gastrointestinal
IBD: Inflammatory bowel disease
ACE2: Angiotensin-converting enzyme 2

## Declarations

### onsent for publication

Written consent was obtained from the patient’s parent for publication of this case report and accompanying images. A copy of the written consent is available for review by the Editor of this journal.
